# A disease progression model estimating the benefit of tolvaptan on time to end-stage renal disease for patients with rapidly progressing autosomal dominant polycystic kidney disease

**DOI:** 10.1186/s12882-022-02956-8

**Published:** 2022-10-18

**Authors:** Gregory Mader, Deirdre Mladsi, Myrlene Sanon, Molly Purser, Christine L. Barnett, Dorothee Oberdhan, Terry Watnick, Stephen Seliger

**Affiliations:** 1 RTI Health Solutions, Research Triangle Park, NC USA; 2 Otsuka Pharmaceutical Development & Commercialization, Inc., Princeton, NJ USA; 3 University of Maryland School of Medicine, Baltimore, MD USA

**Keywords:** Autosomal dominant polycystic kidney disease, Disease modeling, End-stage renal disease, Renal function decline, Tolvaptan

## Abstract

**Background:**

Tolvaptan was approved in the United States in 2018 for patients with autosomal dominant polycystic kidney disease (ADPKD) at risk of rapid progression as assessed in a 3-year phase 3 clinical trial (TEMPO 3:4). An extension study (TEMPO 4:4) showed continued delay in progression at 2 years, and a trial in patients with later-stage disease (REPRISE) provided confirmatory evidence of efficacy. Given the relatively shorter-term duration of the clinical trials, estimating the longer-term benefit associated with tolvaptan via extrapolation of the treatment effect is an important undertaking.

**Methods:**

A model was developed to simulate a cohort of patients with ADPKD at risk of rapid progression and predict their long-term outcomes using an algorithm organized around the Mayo Risk Classification system, which has five subclasses (1A through 1E) based on estimated kidney growth rates. The model base-case population represents 1280 patients enrolled in TEMPO 3:4 beginning in chronic kidney disease (CKD) stages G1, G2, and G3 across Mayo subclasses 1C, 1D, and 1E. The algorithm was used to predict longer-term natural history health outcomes. The estimated treatment effect of tolvaptan from TEMPO 3:4 was applied to the natural history to predict the longer-term treatment benefit of tolvaptan. For the cohort, analyzed once reflecting natural history and once assuming treatment with tolvaptan, the model estimated lifetime progression through CKD stages, end-stage renal disease (ESRD), and death.

**Results:**

When treated with tolvaptan, the model cohort was predicted to experience a 3.1-year delay of ESRD (95% confidence interval: 1.8 to 4.4), approximately a 23% improvement over the estimated 13.7 years for patients not receiving tolvaptan. Patients beginning tolvaptan treatment in CKD stages G1, G2, and G3 were predicted to experience estimated delays of ESRD, compared with patients not receiving tolvaptan, of 3.8 years (21% improvement), 3.0 years (24% improvement), and 2.1 years (28% improvement), respectively.

**Conclusions:**

The model estimated that patients treated with tolvaptan versus no treatment spent more time in earlier CKD stages and had later onset of ESRD. Findings highlight the potential long-term value of early intervention with tolvaptan in patients at risk of rapid ADPKD progression.

**Supplementary Information:**

The online version contains supplementary material available at 10.1186/s12882-022-02956-8.

## Background

Autosomal dominant polycystic kidney disease (ADPKD) is characterized by the formation of renal cysts, resulting in a progressive loss of renal function and, ultimately, end-stage renal disease (ESRD) [1]. ADPKD arises from mutations in *PKD1* and *PKD2* and is the leading genetic cause of ESRD, accounting for 2.5–10% of ESRD cases globally [2–4].

Tolvaptan was approved in the United States (US) in 2018 for patients with ADPKD at risk of rapid progression [5]. Tolvaptan is a vasopressin V2 receptor antagonist demonstrated to slow the progression of cyst development and renal insufficiency in patients with ADPKD [6]. The efficacy and safety of tolvaptan in adults with ADPKD was initially established in a 3-year phase 3 clinical trial (TEMPO 3:4; NCT00428948) [7]. In the open-label extension TEMPO 4:4 trial (NCT01214421), which enrolled 60.3% of the patients in TEMPO 3:4, tolvaptan benefit in terms of slowing decline in estimated glomerular filtration rate (eGFR) was maintained for a further 2 years for patients continuing to receive tolvaptan [8]. An additional study (REPRISE; NCT02160145) [9] was conducted in patients with later-stage ADPKD, further demonstrating treatment efficacy for patients receiving tolvaptan.

Progression of ADPKD is characterized by total kidney volume (TKV) growth, eGFR decline, and subsequent transition to later chronic kidney disease (CKD) stages, including ESRD. Although there are published equations available to predict disease progression [10, 11], accurate statistical modeling can be difficult, mainly due to the underlying sources of substantial variability in the rates of disease progression between patients [12]. The Mayo risk classification system proposed by Irazabal and colleagues [11], which considers baseline age and height-adjusted TKV, has been shown to provide high accuracy in predicting the future rate of progression. The Mayo classification system has five subclasses (1A-1E) characterizing estimated kidney growth rates [11]. Irazabal and colleagues used patient-level data from the Mayo Clinic Translational PKD Center to develop (*n* = 376) and conduct internal validation of (*n* = 162) the prediction equation that incorporates Mayo subclass; the prediction equation also was validated against an external dataset (*n* = 173) from the Consortium for Radiologic Imaging Study of PKD (CRISP) [11].

Patients with rapidly progressing ADPKD can be identified as those in Mayo subclasses 1C, 1D, and 1E [11]. Chebib and colleagues [13] evaluated the use of alternative methods for identifying patients with rapidly progressing ADPKD and recommended use of the Mayo classification system as proposed by Irazabal and colleagues [11]; Yu and colleagues [14] modeled eGFR trajectories from the CRISP dataset and found baseline Mayo subclass to be a strong predictor of eGFR decline; and the European Renal Association–European Dialysis and Transplant Association (ERA-EDTA) Working Groups on Inherited Kidney Disorders and European Renal Best Practice recognized that rapid progression is likely in patients with Mayo subclasses 1C, 1D, and 1E [6]. In addition, the Canadian Working Group recommended use of the Mayo classification system to identify patients at high risk for rapid progression [15]. The ERA-EDTA and Canadian Working Groups also suggest various additional ways to identify risk of rapid progression, such as a kidney length of > 16.5 cm, as assessed by ultrasound [15] in patients < 45 years of age [6]. Importantly, both groups recognize the Mayo classification system as a robust clinical prediction tool [6, 15], and the TEMPO 3:4 clinical trial population was enriched for Mayo subclasses 1C, 1D, and 1E [16].

Researchers have developed cohort models to estimate long-term disease progression for patients with ADPKD [17–19], including a model based on the TEMPO 3:4 clinical trial [19], using a variety of approaches to predict progression. Each of these cohort models can be used to estimate time to ESRD, age at ESRD, and delay of ESRD. However, they assume the same rate of disease progression for all patients; that is, they do not account for rapid progression.

Given the shorter-term duration of the clinical trials, estimating the longer-term benefit associated with tolvaptan treatment of patients at risk for rapid progression is an important undertaking. The objectives of this study were to develop a model to predict long-term natural history health outcomes for a cohort of patients with ADPKD at risk of rapid progression, and to apply the treatment effect of tolvaptan observed in TEMPO 3:4 to the long-term natural history progression to estimate the long-term treatment benefit of tolvaptan. Here, we developed a cohort model, based on Irazabal and colleagues’ [11] equation using the Mayo classification system, to differentiate patients by rate of disease progression.

## Methods

We developed a cohort model with six health states, corresponding to five CKD stages and death, to estimate the long-term natural history health outcomes for patients with ADPKD at risk of rapid progression. Data used to develop this model was collected from previously published studies. This study is not considered research involving human subjects in accordance with the United States Department of Health and Human Services regulation 45 CFR part 46 Subpart A and thus review by an institutional review board was not required. For a cohort of patients with baseline characteristics matching those of patients enrolled in TEMPO 3:4 [7], the model runs twice, first estimating lifetime progression through CKD stages G1 through G5 (ESRD, including no dialysis or transplantation, dialysis, and transplantation) and death for the cohort assuming no treatment, then a second time assuming treatment with tolvaptan, after applying the treatment effect of tolvaptan observed in TEMPO 3:4 [7]. The model then compares the results to estimate the predicted long-term health benefits resulting from treatment with tolvaptan.

### Model structure

**Fig. 1:**
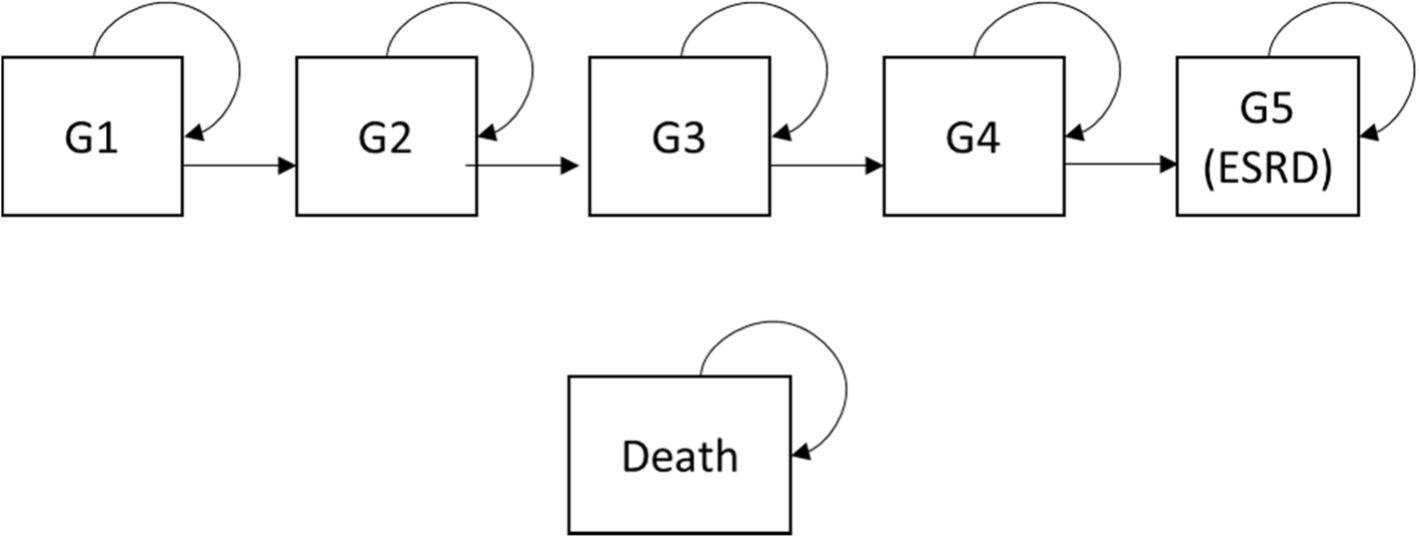
Model Structure for CKD Progression. CKD = chronic kidney disease; ESRD = end-stage renal disease; KDIGO = Kidney Disease Improving Global Outcomes. Note: Patients can reach the death health state from any other health state. G3 includes G3a and G3b. See Additional file 1: Table S1 for a description of CKD stages according to the KDIGO CKD staging system [20]

The model structure (Fig. 1) has six health states, defined by CKD stage G1 through G5 and death. (See Additional file 1: Table S1 for a description of CKD stages according to the Kidney Disease Improving Global Outcomes [KDIGO] CKD staging system [20]). The model generates patient-level progression estimates to simulate a cohort with rapidly progressing ADPKD over a lifetime time horizon. Each year of simulated time, the patients in the cohort are distributed among the health states according to their rates of progression. At the end of each year, patients are reallocated among the CKD stages based on their updated eGFR. Patients can remain in the same CKD stage (e.g., stay in CKD stage G2) or progress to the next consecutive CKD stage (e.g., move from CKD stage G2 to G3); however, patients cannot move to an improved health state (e.g., move from CKD stage G2 to G1).

The model predicts eGFR decline among patients with ADPKD at risk of rapid progression using the Irazabal equation, which estimates eGFR decline as a function of an individual’s baseline age, current eGFR and age-specific, height-adjusted TKV:

$$Future\ eGFR=\alpha +\beta +\gamma \left( baseline\ age\right)+\delta \left( baseline\ eGFR\right)+\theta +\varepsilon \left( years\ from\ baseline\right)+\lambda \left(1\ if\ male,0\ otherwise\right)\left( years\ from\ baseline\right)+\mu \left( current\ age\right)\left( years\ from\ baseline\right)+\sigma \left( years\ from\ baseline\right)$$

The Irazabal equation coefficients for estimating eGFR are shown in Table 1. The estimated annual kidney growth rate for each subclass of rapid progressors are as follows: subclass 1C, 3 to 4.5%; subclass 1D, 4.5 to 6%; subclass 1E, > 6% [11]. Given that rates of progression remain stable in most patients over time (Fig. 6 in Irazabal et al., 2015) [11], the model assumes that patients remain in the same Mayo subclass throughout the lifetime model time horizon. For example, patients classified as 1D progressors move sequentially across CKD stages as their eGFR declines and eventually into ESRD, but 1D progressors never move to 1C or 1E subclasses.

**Table 1:** Irazabal equation coefficients for estimating future eGFR

Variable	Description	Value
α	Intercept	21.18
β	Sex (reference is male)	−1.26
γ	Age at HtTKV0 (years)	−0.26
δ	eGFR at HtTKV0 (mL/min per 1.73 m^2^)	0.90
θc	Subclass 1C	−1.14
θd	Subclass 1D	−1.93
θe	Subclass 1E	−6.26
ε	Years from HtTKV0	−0.23
λ	Sex, years from HtTKV0^a^	0.19
μ	Age at HtTKV, years from HtTKV0^a^	−0.02
σc	Subclass 1C, years from HtTKV0^a^	−2.63
σd	Subclass 1D, years from HtTKV0^a^	−3.48
σe	Subclass 1E, years from HtTKV0^a^	−4.78

Variables pertaining to Subclass 1B are not presented because these patients were not included in the cohort model

Source: Irazabal MV, Rangel LJ, Bergstralh EJ, Osborn SL, Harmon AJ, Sundsbak JL, Bae KT, Chapman AB, Grantham JJ, Mrug M et al.: Imaging classification of autosomal dominant polycystic kidney disease: a simple model for selecting patients for clinical trials. J Am Soc Nephrol 2015, 26(1):160–172

*eGFR* Estimated glomerular filtration rate, *HtTKV* Height-adjusted total kidney volume, *HtTKV0* Baseline height-adjusted total kidney volume

^a^Denotes interaction terms

### Patient population

The TEMPO 3:4 clinical trial (NCT00428948) included 1445 adult patients aged 18 to 50 years with ADPKD with TKV ≥ 750 mL and an estimated creatinine clearance of ≥60 mL/min [7]. The model cohort represented 1280 rapid progressors (Mayo subclasses 1C, 1D, and 1E), regardless of randomization to treatment or placebo, who were enrolled in TEMPO 3:4 beginning in CKD stages G1, G2, and G3 (Table 2).

**Table 2:** Model base-case cohort characteristics

	Males (***N*** = 690)			Females (***N*** = 590)		
	N (% of Total Cohort)	Mean Age (Years)	Mean eGFR (mL/min/ 1.73 m^**2**^)	N (% of Total Cohort)	Mean Age (Years)	Mean eGFR (mL/min/ 1.73 m^**2**^)
CKD stage G1	222 (17.3%)	33.6	105.9	225 (17.6%)	34.5	105.8
Subclass 1C	89 (7.0%)	37.7	105.0	103 (8.0%)	38.6	102.9
Subclass 1D	83 (6.5%)	33.1	102.9	80 (6.3%)	33.8	107.3
Subclass 1E	50 (3.9%)	27.0	112.4	42 (3.3%)	26.0	109.8
CKD stage G2	318 (24.8%)	39.3	74.5	280 (21.9%)	40.1	75.2
Subclass 1C	126 (9.8%)	41.8	74.7	140 (10.9%)	42.5	75.6
Subclass 1D	123 (9.6%)	39.3	74.7	106 (8.3%)	39.1	74.4
Subclass 1E	69 (5.4%)	34.8	74.0	34 (2.7%)	33.0	76.2
CKD stage G3	150 (11.7%)	41.3	50.8	85 (6.6%)	41.7	52.0
Subclass 1C	37 (2.9%)	44.9	52.1	34 (2.7%)	44.6	52.5
Subclass 1D	66 (5.2%)	41.7	51.7	34 (2.7%)	41.7	51.2
Subclass 1E	47 (3.7%)	37.9	48.5	17 (1.3%)	35.8	52.4

Source: Otsuka, data on file (2018). Analysis of baseline data for 1280 typical, rapidly progressing patients enrolled in TEMPO 3:4, regardless of randomization to treatment or placebo

See Additional file 1: Table S1 for a description of CKD stages according to the KDIGO CKD staging system [20]

*CKD* Chronic kidney disease, *eGFR* Estimated glomerular filtration rate

### Effectiveness

For patients receiving tolvaptan, the model applies a constant treatment effect to natural history progression estimates as determined via the Irazabal equation. The annual absolute reduction in eGFR decline for tolvaptan versus placebo of 1.20 mL/min/1.73 m^2^ from TEMPO 3:4 [7] was applied to predicted eGFR decline in the absence of treatment. The model applied the treatment effect for tolvaptan regardless of CKD stage and Mayo subclass level.

### Discontinuation

Consistent with the approach taken by Bennett and colleagues [19], discontinuation rates for the first 3 years of treatment with tolvaptan were based on TEMPO 3:4, and the discontinuation rate in year 4 was assumed to be the same as that in year 3. After year 4, discontinuation was assumed to occur only when patients reached ESRD. A post hoc analysis of TEMPO 3:4 clinical trial data revealed that discontinuation rates were consistent across CKD stages and Mayo subclasses, suggesting that discontinuation is independent of the severity of ADPKD [21]. Thus, the discontinuation rates in the model were assumed to be the same across CKD stages and Mayo subclasses.

### Mortality

Our model estimated the overall survival for patients with ADPKD by applying risk ratios (Table 3) to US general population mortality by age [24]. We calculated risk ratios using the US Renal Data System (USRDS) reported mortality for patients with CKD as a proxy for ADPKD because reliable national-level information on ADPKD-specific mortality was unavailable. The risk ratio was calculated as all-cause mortality for patients with CKD in stages G1 through G5 (excluding patients receiving dialysis or transplantation) divided by all-cause mortality for patients without CKD (using available data, which was for patients ≥66 years of age) and as all-cause mortality for patients receiving dialysis or transplantation divided by all-cause mortality for all Medicare patients (using available data, which was for patients ≥65 years of age).

**Table 3:** Mortality risk ratios

	All-cause mortality per 1000 patient-years	Risk ratio
Patients without CKD	45.6^a^	N/A
Patients with CKD		
CKD stages G1 and G2	82.2^a^	1.80^a^
CKD stage G3	97.2^a^	2.13^a^
CKD stages G4 and G5 (excluding dialysis and transplantation)	181.6^a^	3.98^a^
Dialysis	See USRDS^b^	6.86^b^
Transplantation	See USRDS^b^	2.10^b^

*CKD* Chronic kidney disease; *ESRD* End-stage renal disease, *N/A* not applicable, *USRDS* US Renal Data System

^a^Source: USRDS [22], Fig. 3.2 based on all Medicare patients with CKD ≥ 66 years of age. Values are from 2015. Risk ratios were calculated

^b^Source: USRDS [23], Table 5.5 based on Medicare patients. Values are from 2014 to 2015. Risk ratios were calculated as the average of the mortality risk ratios for patients with ESRD receiving dialysis or transplantation relative to all Medicare. Mortality risk ratios were calculated for males and females, 65–74 years and ≥ 75 years of age

### Validation

The current model’s predictions of ADPKD progression were validated against three published cohort models, none of which relied on the Irazabal equation to estimate progression. Two of the models [17, 18] estimated long-term natural history outcomes for hypothetical individual patients considered representative of various cohorts, including patients enrolled in TEMPO 3:4, as modeled using the ADPKD Outcomes Model (ADPKD-OM) [17, 18]. Bennett and colleagues [19] used the ADPKD-OM to estimate long-term outcomes for the TEMPO 3:4 cohort including both the placebo and tolvaptan arms [19]. To validate the current model’s predictions of ADPKD progression, age at and time to ESRD were compared and the potential influence on the results of the different approaches to modeling ADPKD progression were assessed.

## Results

**Fig. 2:**
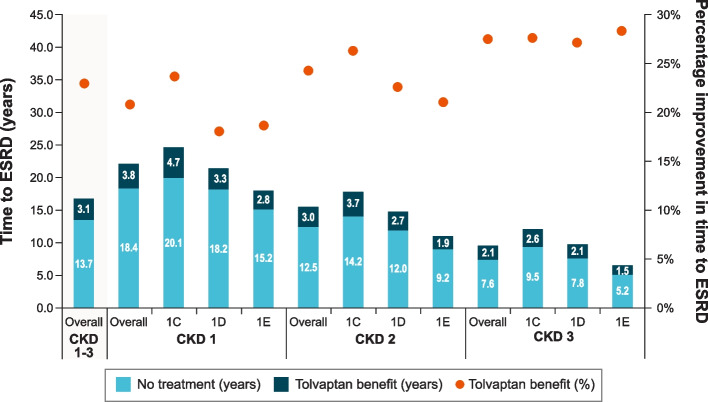
Model-Predicted Benefit of Tolvaptan by CKD Stage and Mayo Subclass on Time to ESRD, CKD = chronic kidney disease; ESRD = end-stage renal disease

Predicted time to ESRD was longer for patients treated with tolvaptan, regardless of Mayo subclass and CKD stage at treatment initiation, with greater benefit predicted the earlier the CKD stage was when treatment was initiated (Fig. 2). Overall, the simulated cohort, which represented the 1280 patients at risk of rapid progression in TEMPO 3:4, was predicted to experience a 3.1-year delay of ESRD (95% confidence interval estimated based on 5000 probabilistic simulations: 1.8 to 4.4) when treated with tolvaptan, a 23% improvement compared with no tolvaptan. When compared with patients not receiving tolvaptan, patients initiating tolvaptan treatment in CKD stages G1, G2, and G3 were predicted to experience an estimated delay of ESRD of 3.8 years (21% improvement), 3.0 years (24% improvement), and 2.1 years (28% improvement), respectively.

**Fig. 3:**
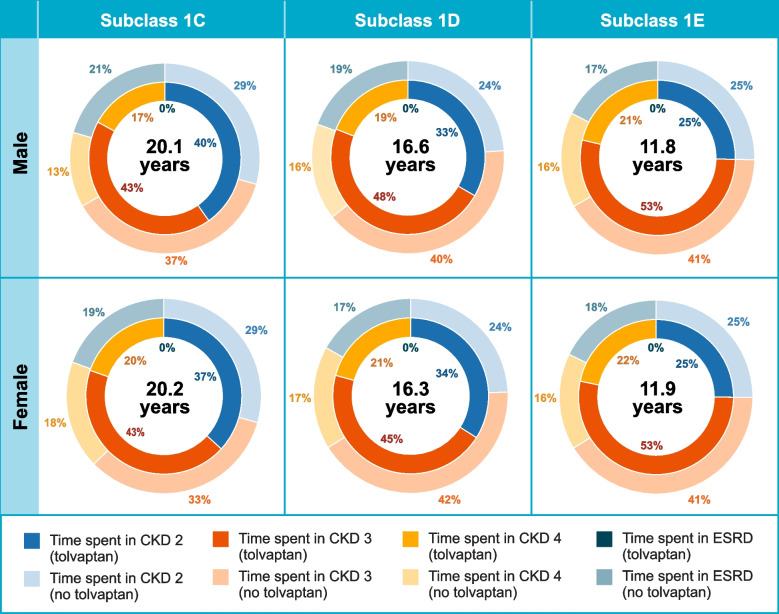
Model Estimates of Time to ESRD for Patients With Tolvaptan and Percentage of Time Spent in Each Health State Over That Time Period for the Average TEMPO 3:4 Patient With Tolvaptan and No Tolvaptan by Mayo Subclass and Sex. CKD = chronic kidney disease; ESRD = end-stage renal disease

Figure 3 shows the predicted time to ESRD by sex by Mayo subclass for the simulated cohort assuming treatment with tolvaptan and shows the percentage of that time spent in each stage of CKD stages G1 through G4. It also shows, for that same time period, the percentage of time spent in each CKD stage (G1 through G5 [ESRD]) assuming no treatment with tolvaptan. For example, predicted time to ESRD for males in Mayo subclass 1C treated with tolvaptan was 20.1 years (Fig. 3). If males in Mayo subclass 1C were not treated with tolvaptan, the model predicted they would have spent 21% of those 20.1 years (4.2 years) in ESRD instead (Fig. 3). For males and females across Mayo subclasses 1C through 1E, the model predicted that 17 to 21% of the years spent without ESRD if treated with tolvaptan would have been spent with ESRD if not treated with tolvaptan (Fig. 3). Predicted time to ESRD with tolvaptan differed by Mayo subclass, but within Mayo subclasses it was similar for males and females (Fig. 3).

Estimated total life years and life years by health state for the model cohort are presented in Table 4. Our cohort model predicted that total life years were increased by 0.7 life years in patients treated with tolvaptan compared with patients not treated with tolvaptan. Additionally, in patients treated with tolvaptan, the model predicted an increase in time spent in non-ESRD CKD stages, and a decrease in time spent in ESRD. Specifically, the model predicted an increase of 0.4 life years spent in CKD stage G1, 1.0 life years spent in CKD stage G2, 1.5 life years spent in CKD stage G3, and 0.3 life years spent in CKD stage G4. Finally, the model estimated a decrease of 2.4 life years spent in ESRD in patients treated with tolvaptan compared with patients not treated with tolvaptan.

**Table 4:** Total life years and life years by health state

	Tolvaptan	No Tolvaptan	Difference^**a**^
Life years^a^	27.4	26.6	0.7
CKD stage G1	1.3	1.0	0.4
CKD stage G2	4.9	3.9	1.0
CKD stage G3	7.3	5.9	1.5
CKD stage G4	3.2	2.9	0.3
ESRD	10.6	13.0	−2.4

*CKD* Chronic kidney disease, *ESRD* End-stage renal disease

^a^Discrepancies in life years and differences are due to rounding

### Validation

Three published models of ADPKD progression were identified and used to validate predictions of natural history progression of ADPKD in our model [17–19]. Erickson and colleagues developed a Markov model of progression through CKD stages [17]. For their base-case analysis, the authors assumed a mean rate of eGFR decline of 3.7 mL/min/1.73 m^2^, which was equal to the annual rate of GFR decline observed in the placebo arm of TEMPO 3:4. Erickson and colleagues [17] assumed variability in the rate of decline based on a large observational study of ADPKD progression (citing Schrier et al., 2003 [25]). Using the model, Erickson and colleagues predicted progression for male and female patients with untreated ADPKD [17]. Presented as representative of the TEMPO 3:4 clinical trial population, the patients were 40 years of age, with an eGFR of 80 mL/min/1.73 m^2^ [17]. Although not specified in Erickson and colleagues [17], the hypothetical patients’ Mayo classifications were probably 1C based on their age and eGFR. Our model estimates of age at ESRD (males, 57 years; females, 56 years) were similar to the results reported by Erickson and colleagues (males, 58 years; females, 57.5 years) [17].

McEwan and colleagues [18] developed the ADPKD-OM, a patient-level simulation that predicts the natural history of ADPKD, using data from the placebo arm of TEMPO 3:4. The authors used it to predict disease progression for hypothetical patient profiles. Although neither the height nor sex of the hypothetical patients were specified, hypothetical patient 1 was approximately 30 years old and was specified to be a 1C or 1D progressor beginning in CKD stage G1, and hypothetical patient 2, who was described by McEwan and colleagues as a rapid progressor, was probably a 1C or 1D progressor based on their age (“late 30s/early 40s”) and baseline TKV (1500 mL) [18]. McEwan and colleagues [18] reported an age at ESRD of 49–54 years for patient 1 and 49–52 years for patient 2; our model estimates for age at ESRD for untreated patients were within those intervals (51.3 years for patient 1; 50.6 years for patient 2).

Bennett and colleagues [19] reported results from the ADPKD-OM for a cohort of patients in Mayo subclasses 1C through 1E (i.e., the patients at risk of rapid progression) matching those enrolled in TEMPO 3:4 in CKD stages G1 through G3 at baseline; the ADPKD-OM was used to estimate the long-term effects of tolvaptan. Bennett and colleagues [19] reported that time to ESRD was 13.0 years for the placebo group (natural history) and 17.3 years for patients in the tolvaptan arm (values were produced by digitizing Fig. 5 in Bennett et al., 2019 [19]), which is a delay of ESRD of 4.3 years. Our model estimated approximately the same number of years to ESRD as the ADPKD-OM under natural history for CKD stages G1 through G3 (13.0 years in ADPKD-OM vs. 13.7 years in our model). Our model compared with the ADPKD-OM estimated a smaller delay of ESRD onset for patients receiving treatment with tolvaptan (3.1 years vs. 4.3 years).

The ADPKD-OM differs from the current model in several key ways, including the approach to estimating eGFR (use of the Chronic Kidney Disease Epidemiology Collaboration [CKD-Epi] equation, whereas our model used the Irazabal equation), the approach for estimating mortality (use of all-cause mortality from the World Health Organization, whereas our model used life tables from the Centers for Disease Control and Prevention with adjusted stage-specific risk ratios estimated with the use of data from the USRDS), the application of treatment discontinuation (assumption of no discontinuation of tolvaptan, whereas our model results assumed discontinuation, upon which treatment effect also discontinued), and the estimate of tolvaptan treatment effect (the tolvaptan treatment effect of 1.11 for rapidly progressing patients [Mayo subclasses 1C, 1D, and 1E] estimated as the difference in annual eGFR slope between patients receiving tolvaptan [− 2.82 mL/min/1.73 m^2^] and patients receiving placebo [− 3.93 mL/min/1.73 m^2^] in TEMPO 3:4, whereas our model applied the overall treatment effect of 1.20 mL/min/1.73 m^2^ from TEMPO 3:4).

## Discussion

Models that differentiate patients by rate of disease progression are needed to estimate the long-term benefit of tolvaptan in patients with ADPKD at risk of rapid progression. In the present study, we developed a model to predict the long-term outcomes of patients with ADPKD, using a cohort of patients at risk of rapid progression (Mayo subclasses 1C, 1D, and 1E). Our cohort model predicted that patients with ADPKD at risk of rapid progression treated with tolvaptan lived longer and spent more time in earlier CKD stages compared with patients not treated with tolvaptan. These findings were consistent across Mayo subclass and CKD stage at treatment initiation and were similar for males and females.

Delay of ESRD is a key goal of ADPKD treatment. Increased healthcare costs [26, 27] and decreased quality of life [28] have been reported for patients entering CKD stage G4 and ESRD, especially costs for those being treated with dialysis or transplantation [29]. Although the ADPKD-OM [19] and our model differ in several important ways, both predict long-term clinical benefit associated with tolvaptan, including a delay to ESRD. Additionally, for a cohort of patients with ADPKD, it is important to account for different rates of disease progression. Unlike other cohort models for ADPKD [17–19], our cohort model differentiated rates of progression by Mayo subclass using a validated risk model recommended for use in clinical care. While our model is not offered as a tool to support individual clinical decision-making, it may be useful for understanding the natural history in a population of patients with ADPKD at risk of rapid progression. Furthermore, these results highlight the potential long-term treatment benefit of early intervention with tolvaptan in this patient population.

Our model includes important limitations regarding its use for estimating the potential long-term benefit of tolvaptan on ADPKD progression. In the absence of longer-term, real-world evidence on the effect of tolvaptan, our model assumed a constant treatment effect and that patients who did not discontinue from treatment before year 4 continued treatment until ESRD. In addition, the model applied the treatment effect of tolvaptan regardless of Mayo subclass and CKD stage. Because no robust mortality data were available for patients with ADPKD at risk of rapid progression, the application of mortality from the general population with CKD may have biased the estimates of age at or time to ESRD. Future studies are warranted to develop disease progression models using ADPKD-specific mortality. Finally, although our model, which used the Irazabal equation, was validated against alternative disease progression models, and the Irazabal equation has been found to be a robust and reasonable approach to predicting disease progression among patients at risk of rapid progression, uncertainty remains regarding the most appropriate long-term modeling approach.

## Conclusions

For patients with ADPKD at risk of rapid progression, our lifetime disease progression model predicted that untreated patients spent less time in the earlier stages of CKD and progressed more rapidly to ESRD compared with patients treated with tolvaptan, who were predicted to spend more time in earlier CKD stages and experience later onset of ESRD. These results were consistent across CKD stages (G1 through G3) and Mayo subclasses (1C through 1E). Given that not all patients with ADPKD progress at the same rate, these results highlight the importance of early intervention with tolvaptan in patients with ADPKD at risk of rapid progression.

## Supplementary Information

**Additional file 1: Supplementary Table S1.** GFR Categories in CKD.

## Data Availability

Otsuka is committed to sharing data in accordance with the EFPIA/PhRMA principles for responsible sharing of clinical trial data guidelines and as required by applicable legislation. Legitimate research requests will be considered. Research proposals requesting patient-level data are reviewed by an Independent Review Panel at WIRB Copernicus Group (https://drc.irbnet.org/release/images/WCG-DRC-Bio.pdf). For inquiries on availability of data of interest, researchers should contact Otsuka (DT-inquiry@otsuka.jp). Please visit https://clinical-trials.otsuka.com/For-Researchers.aspx for further details. Data may be made available following review of a research proposal by the Independent Review Panel at WIRB Copernicus Group for researchers who meet the criteria for access to confidential data.

## Abbreviations

ADPKD: Autosomal dominant polycystic kidney disease
ADPKD-OM: ADPKD Outcomes Model
CKD: Chronic kidney disease
eGFR: Estimated glomerular filtration rate
ESRD: End-stage renal disease
KDIGO: Kidney Disease Improving Global Outcomes
TKV: Total kidney volume
US: United States
