# Supplementary material for: A disease progression model estimating the benefit of tolvaptan on time to end-stage renal disease for patients with rapidly progressing autosomal dominant polycystic kidney disease
Source: BMC Nephrol. 2022 Oct 18;23:334. doi: 10.1186/s12882-022-02956-8 (PMC9578187; doi:10.1186/s12882-022-02956-8)
Supplement: Supplementary file 1 — Additional file 1: Supplementary Table S1. GFR Categories in CKD. [file 12882_2022_2956_MOESM1_ESM.docx]

# Supplemental Appendix

Supplementary Table S1. GFR Categories in CKD

| GFR Category | GFR (mL/min/1.73 m^2^) | Terms |
| --- | --- | --- |
| G1 | ≥ 90 | Normal or high |
| G2 | 60-89 | Mildly decreased |
| G3a | 45-59 | Mildly to moderately decreased |
| G3b | 30-44 | Moderately to severely decreased |
| G4 | 15-29 | Severely decreased |
| G5 | < 15 | Kidney failure |

CKD = chronic kidney disease; GFR = glomerular filtration rate; KDIGO = Kidney Disease Improving Global Outcomes.

Source: Kidney Disease Improving Global Outcomes (KDIGO): Chapter 1: Definition and classification of CKD. Kidney International Supplements. Kidney International Supplements 2013, 3:19-62.
